# High synovial expression of the inhibitory FcγRIIb in rheumatoid arthritis

**DOI:** 10.1186/ar2206

**Published:** 2007-05-23

**Authors:** Sofia E Magnusson, Marianne Engström, Uwe Jacob, Ann-Kristin Ulfgren, Sandra Kleinau

**Affiliations:** 1 Department of Cell and Molecular Biology, Programme for Molecular Immunology, Uppsala University, Husargatan 3, Uppsala, 751 24, Sweden; 2 Department of Medicine, Rheumatology Research Unit, Karolinska University Hospital Solna, Stockholm, 171 76, Sweden; 3 SuppreMol, Am Klopferspitz 19, Martinsried, 821 52, Germany

## Abstract

Activating Fc gamma receptors (FcγRs) have been identified as having important roles in the inflammatory joint reaction in rheumatoid arthritis (RA) and murine models of arthritis. However, the role of the inhibitory FcγRIIb in the regulation of the synovial inflammation in RA is less known. Here we have investigated synovial tissue from RA patients using a novel monoclonal antibody (GB3) specific for the FcγRIIb isoform. FcγRIIb was abundantly expressed in synovia of RA patients, in sharp contrast to the absence or weak staining of FcγRIIb in synovial biopsies from healthy volunteers. In addition, the expression of FcγRI, FcγRII and FcγRIII was analyzed in synovia obtained from early and late stages of RA. Compared with healthy synovia, which expressed FcγRII, FcγRIII but not FcγRI, all activating FcγRs were expressed and significantly up-regulated in RA, regardless of disease duration. Macrophages were one of the major cell types in the RA synovium expressing FcγRIIb and the activating FcγRs. Anti-inflammatory treatment with glucocorticoids reduced FcγR expression in arthritic joints, particularly that of FcγRI. This study demonstrates for the first time that RA patients do not fail to up-regulate FcγRIIb upon synovial inflammation, but suggests that the balance between expression of the inhibitory FcγRIIb and activating FcγRs may be in favour of the latter throughout the disease course. Anti-inflammatory drugs that target activating FcγRs may represent valuable therapeutics in this disease.

## Introduction

Rheumatoid arthritis (RA) is an autoimmune inflammatory disease characterised by autoantibody production and immune complex (IC) formation. Common autoantibodies are rheumatoid factor (RF) and those against citrullinated peptides (CCPs) [1]. Approximately 70% of all RA patients display rheumatoid factor and/or anti-CCP antibodies, and the presence of anti-CCP antibodies can be detected in serum several years before disease debut [2]. Most autoantibodies are of the IgG isotype, which have the potential to activate Fc gamma receptors (FcγRs) on leukocytes, such as macrophages, neutrophils, dendritic cells and B cells. Cross-linking of FcγRs by IgG-ICs leads to cellular effector functions such as phagocytosis, antibody-dependent cellular toxicity and release of inflammatory cytokines.

Three different classes of FcγRs have been identified in humans so far; FcγRI (CD64), FcγRII (CD32) and FcγRIII (CD16). Furthermore, FcγRII and FcγRIII exist in two isoforms, a and b, which carry out divergent functions. FcγRI is a high affinity receptor that binds monomeric IgG as well as IgG-ICs, while FcγRII and FcγRIII are low affinity receptors that predominantly bind IgG-ICs. FcγRI, FcγRIIa, FcγRIIIa and FcγRIIIb are activating receptors. FcγRI and FcγRIIIa consist of an α-chain with three and two Ig-domains respectively, which is connected with a cytoplasmic signalling subunit, the γ-chain. The γ-chain is responsible for intracellular signalling via its immunoreceptor tyrosine based activation motif (ITAM). FcγRIIa is a single chain receptor that contains an ITAM-motif in the cytoplasmic tail. FcγRIIb is an inhibitory receptor that is structurally similar to FcγRIIa, but has an immunoreceptor tyrosine based inhibitory motif in the cytoplasmic domain. FcγRIIb has been shown to have an important negative regulatory function on Fc receptor activation [3].

The involvement of FcγRs in experimental arthritis has been thoroughly investigated, and it is now clear that activating FcγRs are essential for the development of disease. Thus, mice lacking the common γ-chain or FcγRIII are protected from collagen-induced arthritis (CIA) as well as other experimental models of arthritis [4-8]. Consequently, FcγRIIb deficiency in mice leads to increased susceptibility to CIA [9,10]. These findings emphasize the importance of FcγRs in the pathogenesis of experimental arthritis, which may also be true for arthritis in humans. A reported gene polymorphism of FcγRIIIa has been correlated with RA [11-13] as this polymorphism changes the receptor affinity for different IgG-subclasses [14,15]. The FcγRIIIA 158 V/F allele variant has been especially associated with the risk of developing RA [16], although conflicting data exist [17]. Recently, it was also reported that there is an association between rheumatoid factor and the FcγRIIIa 158 V/F allele in RA patients [18] and that a functional variant of FcγRIIb is associated with increased joint destruction in RA but not disease susceptibility [19]. Moreover, several studies have shown that the percentage of FcγRIII positive monocytes is increased in peripheral blood of RA patients [20,21] and that the expression levels of FcγRI, FcγRII and FcγRIII on RA monocytes are increased compared to healthy individuals [22-24], while FcγRIIb expression is unaffected [25].

It has previously been hard to obtain knowledge about FcγR expression in healthy synovial tissue for comparison with FcγR expression in RA patients. Synovia from trauma patients or osteoarthritis patients has often been used as control material [26,27] and only limited studies have been done with healthy synovium [28]. Therefore, we have in this study investigated the expression of the different FcγRs using synovial tissue from healthy volunteers in comparison with RA synovia. We were particularly interested in investigating expression of the inhibitory FcγRIIb as this receptor has not previously been studied due to the lack of a specific antibody against it. Here, by using a novel FcγRIIb specific antibody, GB3, we demonstrate a pronounced FcγRIIb expression in the synovial inflammation in RA, which is in sharp contrast to the lack or weak staining of FcγRIIb in healthy synovia. Additionally, we could detect FcγRII and FcγRIII, but not FcγRI, in healthy synovial tissue. In the RA synovia, the expression of all activating FcγRs was significantly increased, regardless of disease duration. The synovial FcγRI expression could be reduced by intraarticular glucocorticoid treatment.

## Materials and methods

### Subjects

Synovial tissues from 26 RA patients, 12 men and 14 women, were obtained through the rheumatology clinic at the Karolinska Hospital, Solna, Sweden. Characteristics of the patients are presented in Table 1. Healthy synovial biopsies were obtained from ten volunteers, four men and six women, who did not display any arthritic symptoms at the time of the biopsy. An additional group of nine patients with either RA (three men and two women), oligoarthritis (two men) or polyarthritis (two women) was studied before and after treatment with an intraarticular injection of 40 mg of glucocorticoids (triamcinolone hexacetonide; Lederspan, Wyeth Lederle, Solna, Sweden) in the knee. The biopsies were taken before and 9 to 15 days after treatment. The characteristics of these patients are presented in Table 1. All patients included in the glucocorticoid study displayed a decreased synovial inflammation two weeks after treatment as synovial vascularity and hypertrophy were reduced as assessed by arthroscopic evaluation (data not shown). The ethics committee at the Karolinska hospital approved all experiments on human tissue and informed consent was obtained from all study subjects.

**Table 1:** Characteristics of patients

Patient	Category	Diagnosis	RF	Sex/age	Duration of disease	Therapy
1	Early RA	RA	-	M/64	9 mo	None
2	Early RA	RA	+	M/63	3 mo	NSAID
3	Early RA	RA	+	F/77	1.5 mo	None
4	Early RA	RA	ND	M/20	6 mo	NSAID
5	Early RA	RA	+	M/40	11 mo	None
6	Early RA	RA	+	M/54	11 mo	None
7	Early RA	RA	+	F/62	10 mo	NSAID
8	Early RA	RA	+	M/75	18 mo	NSAID
9	Early RA	RA	+	M/82	12 mo	NSAID
10	Early RA	RA	+	M/51	11 mo	NSAID
11	Early RA	RA	+	F/76	12 mo	Salazopyrin
12	Late RA	RA	-	F/47	20 yr	MTX
13	Late RA	RA	+	F/67	40 yr	MTX
14	Late RA	RA	+	F/62	12 yr	MTX, NSAID
15	Late RA	RA	+	F/65	30 yr	MTX, NSAID, Pred
16	Late RA	RA	+	F/63	2 yr	MTX, NSAID, Pred
17	Late RA	RA	+	F/83	11 yr	MTX, Pred
18	Late RA	RA	+	M/38	22 yr	Enbrel, MTX
19	Late RA	RA	+	F/72	44 yr	Cyclosporine, NSAID
20	Late RA	RA	+	F/59	18 yr	Salazopyrin, Glucosamine
21	Late RA	RA	+	M/73	20 yr	Paracetamol
22	Late RA	RA	+	F/59	11 yr	MTX, Remicade
23	Late RA	RA	+	M/69	40 yr	MTX, Pred, Cyclosporine
24	Late RA	RA	+	M/53	7 yr	Pred, NSAID
25	Late RA	RA	+	F/55	4 yr	Remicade
26	Late RA	Juvenile RA	+	F/35	20 yr	MTX, NSAID
27	Glu-study	RA	+	M/53	7 yr	Pred, NSAID
28	Glu-study	RA	+	M/63	3 mo	NSAID
29	Glu-study	RA	-	F/35	20 yr	MTX, NSAID
30	Glu-study	Oligoarthritis	-	M/21	4 mo	None
31	Glu-study	Oligoarthritis	-	M/53	10 yr	None
32	Glu-study	Polyarthritis	-	F/43	12 yr	Pred, NSAID
33	Glu-study	Polyarthritis	+	F/73	2 yr	MTX
34	Glu-study	RA	ND	M/20	6 mo	NSAID
35	Glu-study	RA	+	F/55	4 yr	Remicade

Glu-study, before and after glucocorticoid study; F, female; M, male; mo, month; MTX, methotrexate; ND, not determined; NSAID, non-steroidal anti-inflammatory drugs; Pred, prednisolone; RA, rheumatoid arthritis; RF, rheumatoid factor; yr, year.

### Synovial tissue

The synovial membrane biopsies were obtained by an arthroscopic technique as previously described [29] and were taken, when possible, from synovitis adjacent to the cartilage-pannus junction. Synovial tissue was also obtained at articular surgery in late RA cases. The synovial tissues were divided into three groups: I, healthy (*n *= 10); II, early RA, diagnosed for less than 18 months (*n *= 11); and III, late RA, diagnosed for more than 18 months (*n *= 15). The biopsies from glucocorticoid treated patients (*n *= 9) were grouped into before and after treatment.

### Tissue preparation

The tissue was snapfrozen in liquid isopentane chilled with dry ice and stored at -70°C until sectioned. Frozen biopsies were embedded in OCT compound (TissueTek, Sakura Finetek, Zoeterwoude, The Netherlands) and sectioned into 7 μm serial sections using a cryostat onto SuperFrost Plus slides (Menzel-Gläser, Braunschweig, Germany). The slides were air dried for 30 minutes, then fixed for 20 minutes at 4°C with 2% (volume/volume) formaldehyde (Sigma, St Louis, MO, USA) in PBS, pH 7.4, then washed in PBS and left to air dry and stored at -70°C until use.

### Staining of cell lines

The GB3 (mouse IgG1) monoclonal antibody (mAb), specific for human FcγRIIb, was generated by immunization of mice with a recombinant CD32b coupled cyclic 126-SKKFSRSDPNFSG-138 peptide, including N-acetyl-glucosaminylated asparagine at position 135. The cyclic peptide represents a loop in the Fc-fragment binding region of CD32 that carries unique residues for the inhibitory b-form of the receptor and should facilitate a specific immune response against the CD32b unique glycosylation site at Asn135 of the receptor. Mice were sacrificed and splenic B-cells were fused with immortalized cells using standard protocols. The propagation of approximately 700 hybridoma clones resulted in the GB3 clone. To test the specificity of the GB3 mAb, the monocytic U937 cell line (kind gift from Prof. Lars Hellman, Uppsala University, Sweden) and the B cell lymphoma Raji cell line (kind gift from Dr Fredrik Öberg, Uppsala University, Sweden) were stained with the GB3 mAb, mouse IgG2b anti-FcγRIIa (clone IV.3; kind gift from Dr Johan Rönnelid, Uppsala University, Sweden), mouse IgG1 pan anti-human FcγRII (clone KB61, DAKO, Glostrup, Denmark) or isotype controls (mouse IgG1, DAKO and mouse IgG2b, Sigma) for 30 minutes at 4°C. The cells were then washed in 1% BSA in PBS and a phycoerythrin-(PE-)conjugated rabbit anti-mouse IgG secondary antibody (Biosite, Täby, Sweden) was added to the cells and incubated for 30 minutes at 4°C. After further washing, the cells were re-suspended in 1% BSA in PBS and analysed using a FACScan (Becton Dickinson, Mountain View, CA, USA).

### ELISA

To confirm the specificity of the GB3 mAb, enzyme-linked immunosorbent assay (ELISA) was used. Microtiter plates (Immunolon 2 HB, Dynex Technologies Inc., Chantilly, VA, USA) were coated with 2 μg per well of recombinant soluble (s)FcγRIIb or recombinant sFcγRIIa (both R&D systems, Minneapolis, Minnesota, USA) diluted in 0.5% BSA in PBS and incubated overnight at 4°C in a humid chamber. The plate was then washed with 0.05% Tween 20 (Merck, Schuchardt, Germany) in PBS. The epitope specificity of the GB3 mAb was determined by titrating the antibody five times in each step, with a starting concentration of 5 μg/ml in 0.5% BSA, and then serially added to the plate in duplicates and incubated for 2 h at room temperature (RT). The plate was washed and 50 μl of sheep anti-mouse IgG conjugated to alkaline phosphatase (Sigma) was added per well and incubated for 2 h at RT. The plate was washed and 50 μl per well of *p*-nitrophenyl phosphate substrate (1 mg/ml; Sigma) in ethanolamine buffer were added and the plate incubated in the dark. The absorbance value was determined at 405 nm in an ELISA reader (Molecular Devices Corporation, Sunnyvale, CA, USA).

### Immunohistochemistry

Slides were thawed and washed in PBS with 0.1% saponin (PBS/Sap, pH 7.4) for 10 minutes. Any endogenous peroxidase was blocked using 1% H_2_O_2 _in PBS/Sap for 1 h at RT in dark. Sections were then washed repeatedly in PBS/Sap. The primary antibody, diluted in PBS/Sap, was added and left at RT over night in the dark. After several washing steps, the sections were incubated with 1% normal horse serum in PBS/Sap for 15 minutes. The serum was thereafter removed and biotinylated horse anti-mouse secondary antibody, diluted in 1% normal horse serum, was added for 30 minutes at RT. Sections were washed and avidin-biotin-complex (ABC-elite, Vectastain elite kit, Vectorlab, Burlingame, CA, USA) added for 45 minutes according to the manufacturer's instructions. Subsequently, after washing, any positive staining was developed in diaminobenzidine (DAB; DAB substrate kit, Vectorlab) for 7 minutes according to the manufacturer's instructions. Finally, the sections were counterstained with haematoxylin (Histolab, Gothenburg, Sweden), rinsed with tap water, dehydrated in alcohol and mounted with Mountex (Histolab).

### Immunofluorescent staining

Formaldehyde fixed sections were washed in PBS/Sap and incubated with 0.1% BSA in PBS/Sap for 30 minutes at RT. If necessary, 20% normal human serum was added for 30 minutes at RT to block any non-specific binding of antibody before incubation with 0.1% BSA in PBS/Sap. Fluorescently labelled or unlabelled primary antibody was then diluted in PBS/Sap with 0.1% BSA and added for 90 to 120 minutes at RT followed by washing in either PBS or PBS/Sap. For non-conjugated antibodies, biotinylated or fluorescently labelled secondary antibody was added for 30 minutes followed by washing in PBS/Sap. Then streptavidin conjugated to either alexa-red or green was added for one hour when needed. Sections were then washed in PBS, dried and mounted in PBS/glycerol or Mowiol (Calbiochem, San Diego, CA, USA).

### Primary antibodies

A commonly used marker for macrophages is the CD68 antigen. However, this marker can also be found on fibroblasts and other leukocytes depending on the mAb clone used for detection [30,31]. So to avoid this and exclusively investigate mature monocytes/macrophages as a source of FcγR expression, we used a mAb against CD163. The CD163 antigen is known to be exclusively expressed by mature peripheral blood monocytes and macrophages [32,33]. The antibodies used were thus PE-conjugated anti-CD163 (clone 215927; R&D-systems), non-conjugated anti-CD163 (clone Ber-MAC3; DAKO), FITC-conjugated and non-conjugated anti-CD64 (clone 10.1; BD Pharmingen, San Diego, CA, USA), FITC-conjugated and non-conjugated anti-CD32 (clone KB61; DAKO), non-conjugated anti-CD32b (clone GB3), FITC-conjugated and non-conjugated anti-CD16 (clone DJ130c) (DAKO), non-conjugated anti-CD3 (clone SK7; Becton Dickinson), non-conjugated anti-CD19 (clone HD37; DAKO) and non-conjugated anti-CD20 (clone L27; Becton Dickinson). All antibodies were of mouse IgG1 isotype and an irrelevant mouse IgG1 (DAKO) was used as negative control. As an additional specificity control, the GB3 mAb was absorbed by incubating the GB3 mAb with recombinant human FcγRIIb protein at a 1:1 ratio overnight before adding it to the tissue sections.

### Secondary antibodies

Secondary antibodies included biotinylated horse anti-mouse IgG (Vector, Burlingame, CA, USA), FITC-conjugated F(ab')_2_-fragment of rabbit anti-mouse immunoglobulins (DAKO) and PE-conjugated rabbit anti-mouse IgG secondary antibody (Biosite).

### Microscopic analysis

The immunohistochemical staining was analysed in a Polyvar II light microscope (Reichert-Jung, Vienna, Austria) and evaluated by two independent observers (SEM and SK). Both observers were blinded to the tissue identity and staining. An arbitrary scale was used to identify the amount of positively stained area of the whole tissue section, where 0 = 0% positive tissue area, 1 = 1% to 20% positive tissue area, 2 = 21% to 50% positive tissue area, 3 = 51% to 80% positive tissue area and 4 = 81% to 100% positive tissue area. The staining pattern was also noted as well as presence of vessels and lymphocyte infiltrates. The immunofluorescence staining was analysed using a Leica DMRXA2 fluorescence microscope (Leica Microsystems, Cambridge, UK).

### Statistical analyses

The Mann-Whitney rank sum test was used on unpaired immunohistochemistry data, the Wilcoxon signed rank test on paired immunohistochemistry data and correlations were determined using Spearman's rank correlation.

## Results

### Specificity of the GB3 anti-FcγRIIb mAb

**Figure 1:**
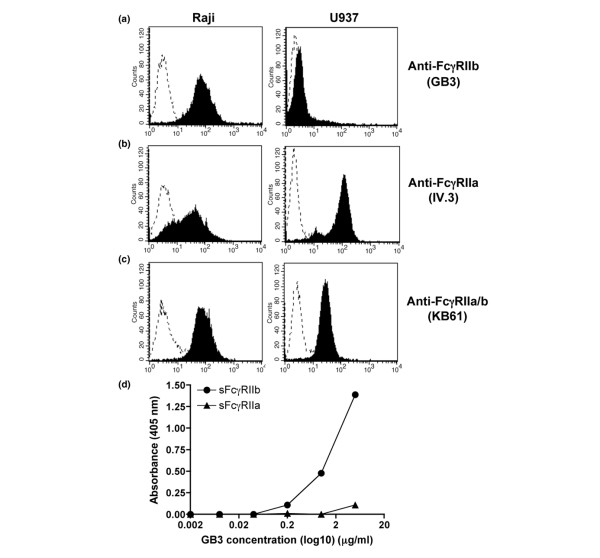
The monoclonal antibody (mAb) GB3 is specific for human Fc gamma receptor (FcγR)IIb. **(a-c) **Human B cell (Raji) or monocyte (U937) cell lines were stained with either the anti-FcγRIIb mAb GB3, the anti-FcγRIIa mAb IV.3 or the pan anti-FcγRII mAb KB61. Filled graphs represent the specific antibody and dotted graphs the isotype control antibody. Raji cells were clearly stained positive for FcγRIIb by the GB3 mAb, while U937 cells were negatively stained, thus demonstrating the FcγRIIb specificity of the GB3 mAb (a). Raji cells were, somewhat surprisingly, stained positive by IV.3 mAb and, as expected, so were the U937 cells (b). This indicates that the IV.3 mAb detects FcγRIIa but is also cross-reactive to FcγRIIb. Both cell lines were positively stained by the pan anti-FcγRII mAb, detecting both the a and b isoforms (c). **(d) **The epitope specificity of the GB3 mAb as demonstrated by ELISA. Serially diluted GB3 mAb show binding activity to recombinant sFcγRIIb whereas no reactivity to recombinant sFcγRIIa.

To verify the specificity of the novel GB3 mAb against FcγRIIb, U937 cells positive for FcγRIIa [34] and Raji cells positive for FcγRIIb [35] were stained with the GB3 mAb or the previously described IV.3 mAb specific for FcγRIIa [34]. The GB3 mAb showed strong positive staining of the Raji cells, whereas the U937 cells were stained negative (Figure 1a). In contrast, the IV.3 mAb clearly stained the U937 cells while the Raji cells were partly stained (Figure 1b), indicating that the IV.3 mAb is somewhat cross reactive with FcγRIIb, as has recently also been reported [35]. Both cell lines were clearly stained by the KB61 pan anti-FcγRII mAb, showing detection of both FcγRIIa and FcγRIIb expression (Figure 1c). The epitope specificity of the GB3 mAb was also determined by ELISA. The ELISA data show that the GB3 mAb binds recombinant sFcγRIIb protein, but not recombinant sFcγRIIa protein (Figure 1d). These results demonstrate that the GB3 mAb is specific for FcγRIIb and that it distinguishes between FcγRIIa and FcγRIIb.

### Strong synovial FcγRIIb expression in RA patients

**Figure 2:**
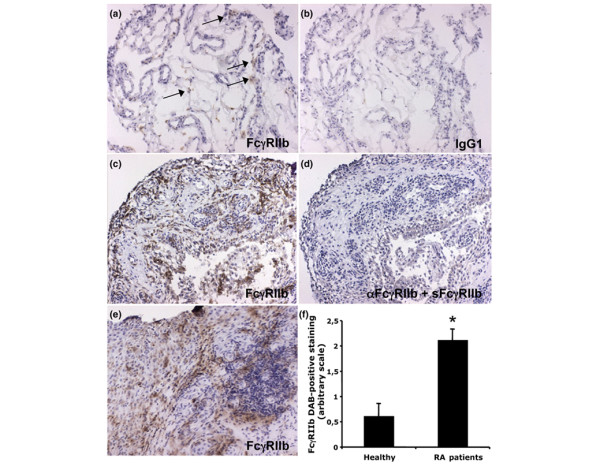
Expression of Fc gamma receptor (FcγR)IIb in healthy and rheumatoid arthritis (RA) synovia. (a) FcγRIIb was sparsely expressed in the few healthy synovial biopsies that were positively stained by GB3. The arrows indicate FcγRIIb positive cells in the sub-lining synovial layer and tissue. (b) The IgG1 isotype control antibody did not stain the healthy synovium. (c,e) Positive FcγRIIb expression was found in all the stained RA synovial biopsies. FcγRIIb staining was observed in the synovial lining and sub-lining layers, perivasculary and inside lymphocyte infiltrates (e). (d) No staining of the RA synovium was observed when the anti-FcγRIIb monoclonal antibody was bound by recombinant human soluble FcγRIIb prior to staining. (f) Synovial FcγRIIb expression was significantly up-regulated in RA patients (*n *= 10) compared to healthy individuals (*n *= 5) (mean values of two independent observers, SEM and SK with standard error of the mean, *p = 0.0104). DAB, diaminobenzidine. (Original magnification ×125.)

Expression of the inhibitory FcγRIIb in healthy and RA synovial tissue was investigated using the GB3 mAb described above. In healthy synovia (*n *= 5), FcγRIIb was either not detected, or only weakly expressed in some individuals. The few biopsies that were positive for FcγRIIb displayed weak positive staining in the sub-lining layer and in scattered cells of the synovia (Figure 2a). The IgG1 isotype control antibody did not stain the tissue (Figure 2b). The RA synovial tissue, however, displayed quite strong FcγRIIb expression, which was evident in all investigated patients (*n *= 10; Figure 2c,e). FcγRIIb was found in the synovial lining layer and in the sub-lining layers of the synovium (Figure 2c). FcγRIIb expression was also found perivasculary to some extent and in scattered cells throughout the tissue. Some staining of FcγRIIb was also observed within lymphocyte infiltrates (Figure 2e). Thus, when consecutive slides were stained with an anti-CD19/CD20 mAb mix, anti-FcγRII and anti-FcγRIIb, there were overlapping staining patterns between these markers, indicating that B cells in the RA synovia express FcγRIIb (data not shown). No staining was observed when the GB3 mAb was bound by recombinant human sFcγRIIb (Figure 2d) or when staining with an IgG1-isotype control antibody (data not shown). RA synovial FcγRIIb expression was significantly increased compared with FcγRIIb expression in healthy synovia (*p *= 0.0104; Figure 2f). This indicates that there is a greater need for negative regulation of the activating FcγRs in arthritic joints than in healthy joints.

### Augmented expression of activating FcγRs in RA synovia

**Figure 3:**
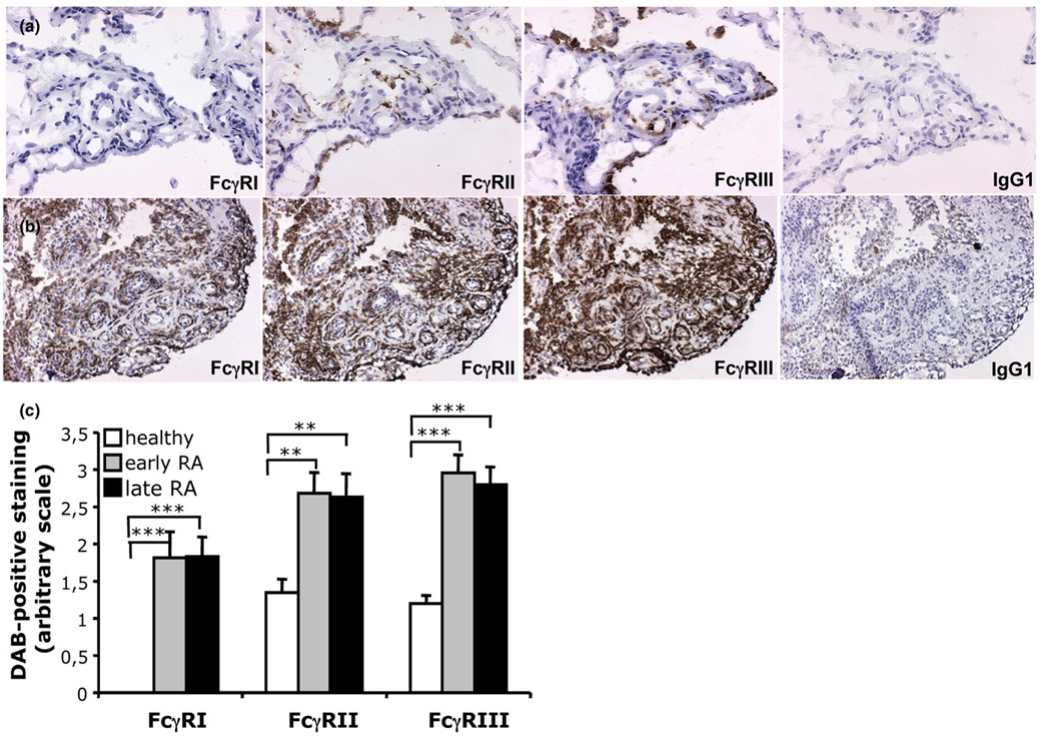
Increased Fc gamma receptor (FcγR)I, II and III expression in rheumatoid arthritis (RA) synovia. **(a) **Sequentially sectioned synovial biopsy from a healthy individual demonstrating FcγRII and III, but not FcγRI, positive cells. The same expression pattern was seen in all healthy volunteers studied (*n *= 10). (Original magnification ×250.) **(b) **Sequentially sectioned synovial tissue from a RA patient demonstrating FcγRI, II and III positive cells in the synovial lining and sub-lining layers and perivasculary. Note the dense staining of FcγRIII in the synovial lining layer. The FcγR stainings were representative of all other RA tissues stained (*n *= 26). IgG1 isotype control stainings were included for all the patient material used. (Original magnification ×125.) **(c) **The expression of FcγRs in healthy (*n *= 10), early (*n *= 11) and late RA (*n *= 15) synovial tissue was evaluated and compared. Note that FcγRI was not detected in healthy synovial tissue and that FcγRI, FcγRII and III were significantly more expressed in both early and late RA synovial tissue compared to healthy synovia (mean values of two independent observers, SEM and SK with standard error of the mean; **p < 0.01; ***p < 0.001). DAB, diaminobenzidine.

As no thorough analysis of the expression of the activating FcγRs in healthy synovia has previously been performed we set out to analyse synovial biopsies from 10 healthy volunteers to compare them with synovia from RA patients (*n *= 26). In addition, to investigate if FcγR expression differs in newly diagnosed RA compared with FcγR expression in late stages of RA, the RA patients were divided into early (*n *= 11) and late RA (*n *= 15) depending on how long they had been diagnosed with RA. All biopsies were stained with mAbs against FcγRI, FcγRII and FcγRIII. The pan KB61 anti-FcγRII antibody was used to analyse the combined expression of FcγRIIa and IIb, as no truly specific anti-FcγRIIa antibody is available. The previously described anti-FcγRIIa antibody (IV.3) is not able to clearly distinguish between FcγRIIa and IIb, as was demonstrated in our stainings of cell lines (Figure 1b). The staining of healthy synovia (Figure 3a) revealed a novel finding in that FcγRI expression could not be detected in any of the biopsies studied. Expression of FcγRII and III was observed, however, most often in the synovial lining layer and in the sub-lining layer. However, FcγRIII was not detected as often as FcγRII in the sub-lining layer. Both FcγRII and III were occasionally detected perivasculary when vessels were present. Staining of healthy synovia with IgG1 isotype control antibody did not show any background staining. In contrast, RA synovium showed high expression of all FcγRs (Figure 3b). FcγRI was observed perivasculary and in some cases also in the synovial lining and sub-lining layers; however, FcγRI positive cells were rarely seen within lymphocyte infiltrates. The expression pattern of FcγRII and III was very similar and both receptors were often found perivasculary and always in the synovial lining and sub-lining layers of the synovium. FcγRII and III positive cells were also observed within lymphocyte infiltrates. The IgG1 isotype control antibody was negative in most RA tissues but showed weak background staining in some of the RA patients, possibly due to FcγR interactions (Figure 3b). Furthermore, as for FcγRIIb, the percentages of FcγRI, II or III positive cells were significantly greater in RA synovial tissue compared to healthy synovial tissue (Figure 3c). We did not find any significant differences between the early and late RA patients with respect to synovial FcγR expression.

### FcγR expressing macrophages in RA synovium

**Figure 4:**
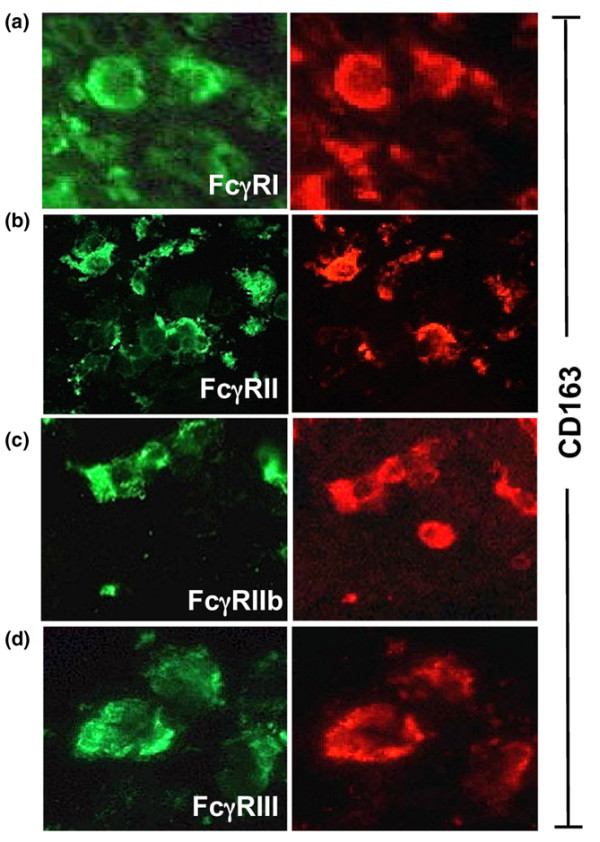
Co-localisation of CD163 with Fc gamma receptor (FcγR)I, II, IIb and III. **(a-d) **Rheumatoid arthritis synovial tissue showed overlapping expression of the macrophage marker CD163 with the expression of FcγRI (a), FcγRII (b), FcγRIIb (c) and FcγRIII (d). The majority of the CD163 positive macrophages expressed FcγRIIb. However, note that a CD163 positive cell lacked FcγRIIb expression. (Original magnification ×400.)

**Figure 5:**
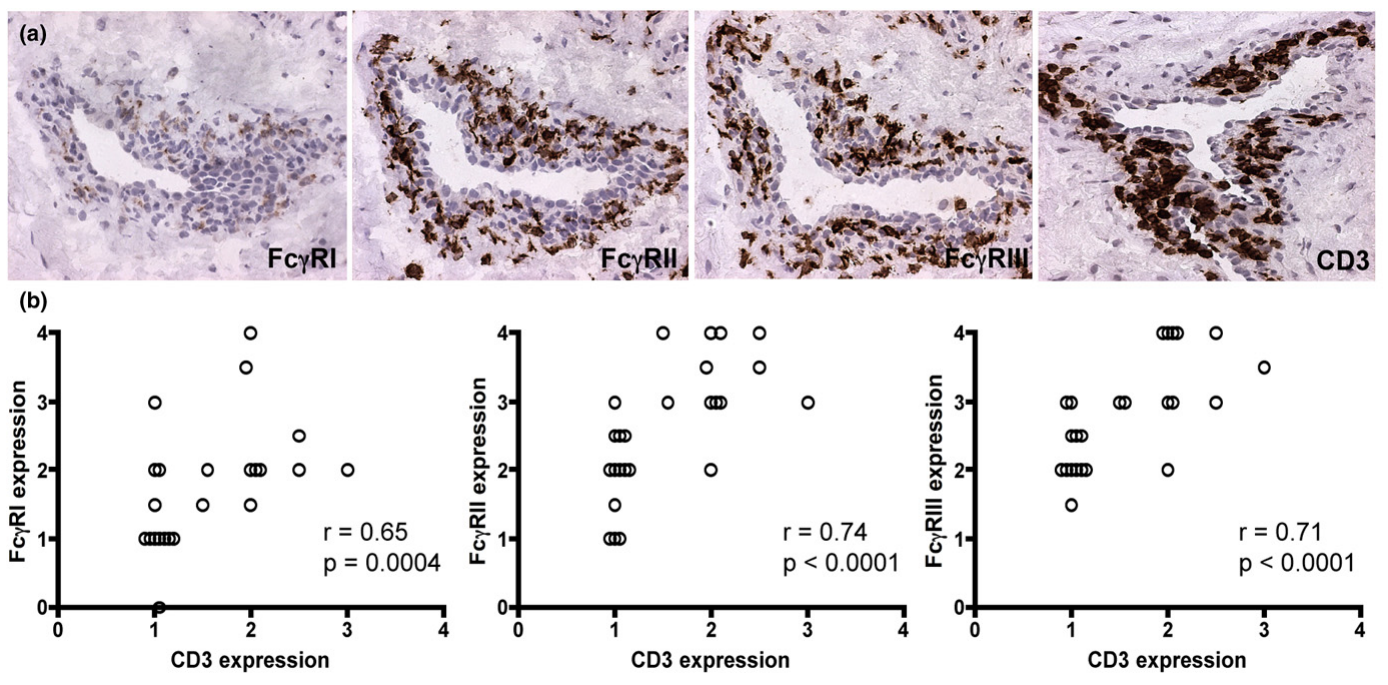
Fc gamma receptor (FcγR) expression correlates with the presence of T cells in rheumatoid arthritis (RA) synovium. **(a) **Sequentially sectioned synovial tissue from a late stage RA patient demonstrating FcγRI, II and III positive cells perivasculary. The FcγR positive cells are located in the same area as CD3 positive T cells. Note the FcγRIII positive cell inside the vessel. This staining was representative of all other late RA synovial tissues stained in the same way (*n *= 26). The IgG1 isotype control did not show any background (data not shown). (Original magnification ×250.) **(b) **The expression of FcγRI, II and III was significantly correlated with the expression of CD3 in RA synovium (each symbol represents one patient, *n *= 26).

To investigate if macrophages were one of the major FcγR expressing cells in the RA joints, RA synovia was double stained with a PE-labelled anti-CD163 (a macrophage specific marker) mAb and an alexa green-labelled anti-FcγRI, FcγRII, FcγRIIb or FcγRIII mAb. The staining revealed that RA macrophages indeed were positive for the inhibitory and activating FcγRs, as demonstrated by the clear expression of FcγRI, FcγRII, FcγRIIb and FcγRIII on CD163 positive cells (Figure 4a–d). Interestingly, even though all RA synovial biopsies showed double staining for CD163 and FcγRIIb, some CD163 positive cells did not express FcγRIIb (Figure 4c). This suggests that there might be a FcγRIIb negative subpopulation of macrophages that are not able to regulate their FcγR activity in the RA synovium. Biopsies stained with PE or alexa green-conjugated control antibodies were negative (data not shown). Moreover, consecutive DAB stained slides of CD163 positive macrophages and FcγR positive cells showed that the amount of FcγRI, II and III expression was significantly correlated with CD163 expression in the RA synovia (FcγRI, r = 0.65, *p *= 0.012; FcγRII, r = 0.85, *p *= 0.0001; FcγRIII, r = 0.75, *p *= 0.002), which confirms the results of the immunofluorescence stainings. Furthermore, we observed that FcγR-positive cells, most likely macrophages, often co-localised with CD3-positive T cells around vessels of the synovial tissue (data not shown). Thus, a significant correlation was found between the expression of FcγRI, II and III and that of CD3 in RA patients, indicating the presence of FcγR positive macrophages near T cells in RA synovia (Figure 5).

### Local anti-inflammatory treatment reduces synovial FcγR expression

**Figure 6:**
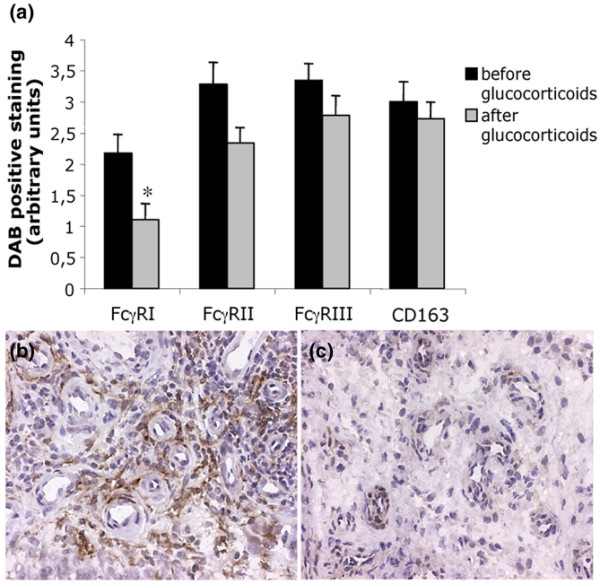
Decreased Fc gamma receptor (FcγR) expression after local treatment with glucocorticoids. **(a) **Immunohistochemical staining of FcγRs and macrophages (CD163) in arthritic synovial tissue (*n *= 9) before and after local glucocorticoid treatment of the joint was analysed. The FcγRI expression was significantly decreased after treatment (*p < 0.05) and there was a trend towards decreased expression of FcγRII (*p *= 0.074). **(b,c) **Staining of FcγRI on arthritic synovial biopsy, taken before and after one intraarticular injection of glucocorticoids, from the same biopsy area and patient. Note the reduced FcγRI staining after treatment (c). DAB, diaminobenzidine.

Glucocorticoid treatment is often successfully used to suppress inflammation in arthritic joints. Therefore, we wanted to investigate whether an intraarticular injection of glucocorticoids has any effect on synovial FcγR expression. Synovial biopsies from the knee of RA, polyarthritic and monoarthritic patients were stained for individual FcγRs before and after glucocorticoid treatment. The expression of FcγRI was significantly reduced two weeks after the glucocorticoid injection (*p *= 0.027; Figure 6a). The decrease in FcγRI expression was clearly seen in the sub-lining and lining layers and, in particular, perivasculary (Figure 6b,c). There was also a trend to decreased FcγRII expression, although this did not reach significance (*p *= 0.074). The presence of CD163 positive macrophages was also investigated before and after glucocorticoid treatment but no difference in CD163 expression was found. No background staining was seen using an isotype control antibody on tissue from all patients, before and after treatment (data not shown).

## Discussion

These data demonstrate that the inflammation in RA synovium is characterised by a pronounced expression of the inhibitory FcγRIIb, which suggests it has a role in counteracting the effects of activating FcγRs in RA synovia. Specific FcγRIIb expression patterns in RA or healthy synovia have not previously been described; nor has the expression of activating FcγRs in healthy synovia been described fully. Synovium from trauma patients has been reported to express FcγRI, II and III, and the FcγRII and III expression has been shown to be significantly lower in these patients compared to RA patients, while their FcγRI expression does not differ from that of RA synovia [27]. This is in contrast to our findings where we could not identify any FcγRI expression in healthy synovium. Thus, it appears that FcγRI is expressed as a consequence of a general inflammation in the joint, as we also observed FcγRI expression in synovial tissue from osteoarthritis patients (data not shown), although not to the same extent as seen in RA patients. The fact that FcγRI is absent from healthy synovial tissue but present in RA synovium and significantly decreased by administration of glucocorticoids indicates that FcγRI has a significant inflammatory role in the pathogenesis of arthritis. This is interesting and in line with experimental studies where FcγRI deficiency in mice leads to a reduced uptake of IgG-ICs and to decreased IC-induced inflammation [36]. It has also been reported that up-regulation of FcγRI leads to increased cartilage destruction in arthritic mice [37]. Similar to FcγRI, FcγRIIb was absent from, or only weakly expressed in, healthy synovia, whereas RA synovium clearly expressed FcγRIIb. This indicates a need for FcγRIIb to control the stimulatory activity of the ITAM-containing receptors in the RA joint.

Although the majority of RA macrophages expressed FcγRIIb, it was also evident that some macrophages did not. This may point towards a small subpopulation of FcγRIIb negative macrophages that may have extraordinary inflammatory capacities. We also observed FcγRIIb expression in lymphocyte infiltrates of RA synovium, which further suggests that infiltrating B cells may also be regulated by this receptor. In contrast to the near absence of FcγRIIb expression in healthy synovia, we clearly observed positive FcγRII staining with the pan anti-FcγRII mAb, most likely as a result of the presence of the activating FcγRIIa in healthy synovia. FcγRIII is also expressed in healthy synovia, ready to bind ICs caught in the joint. In RA synovium both FcγRII and FcγRIII expression was significantly increased, as has also previously been observed [26-28]. However, no association with disease duration and the degree of FcγR expression in RA patients could be shown, as both early and late patient groups expressed similar amounts of FcγRs. This suggests that the FcγRs are important during the whole disease course and not only in the induction phase of RA.

The importance of FcγRII and FcγRIII in arthritis has also been emphasized in animal models of RA. Transgenic mice expressing human FcγRIIa [38] develop CIA much earlier than wild-type mice and normally arthritis resistant mice become susceptible to arthritis when expressing FcγRIIa [39]. Furthermore, the induction of CIA is dependent on FcγRIII and, in particular, FcγRIII positive macrophages [4,40]. Studies of RA monocytes/macrophages have also stressed the significance of FcγRII and FcγRIII in disease pathogenesis. Thus, FcγRII and FcγRIII are up-regulated on peripheral blood monocytes and FcγRIII expression is also enhanced on synovial fluid macrophages [21,22].

The importance of FcγRII in RA was also noted in this study by the decrease in FcγRII expression (albeit not significant) after glucorticoid treatment. The decrease in FcγRII as well as FcγRI expression after local glucocorticoid injection was not due to a reduced number of macrophages in the tissue, as no significant difference in CD163 expression was found after treatment. In agreement, a previous study, where the same patient material was included, also found that the amount of CD163 and CD68 positive cells were not affected by intraarticular glucocorticoid treatment [41]. This indicates that FcγRs are down regulated from the cell surface by glucocorticoid treatment, which may help to explain some of the improvement seen in RA patients upon treatment with it, in addition to its reported suppressive effects on cytokines [41]. These results are in line with an earlier report that indirectly showed that FcγR expression is decreased on peripheral blood monocytes from autoimmune hemolytic anemia patients after systemic administration of corticosteroid, measured by radiolabelled IgG-binding [42]. In a more recent paper, FcγRI and II were shown to be decreased on peripheral blood monocytes one month after systemic therapy with daily low glucocorticoid doses [22]. Recent studies with other anti-rheumatic drugs have demonstrated that methotrexate treatment could reduce the expression of FcγRI and IIa on peripheral blood monocytes from RA patients, while the expression of FcγRIII was unaffected [43]. We did not see a clear reduction in FcγRIII expression after glucocorticoid treatment, which might indicate that FcγRIII expression is hard to modify using anti-rheumatic drugs. It is difficult to speculate on how relevant the reduction of FcγRs is for the physiological outcome of glucocorticoid treatment, since patients receiving an intraarticular injection of glucocorticoids experience an almost instant effect, but positive anti-inflammatory effects in the joint are seen several weeks after treatment.

Macrophages are present in the synovial lining layer of healthy synovium and the amount of macrophages in joints of RA patients is correlated with disease activity [44-46]. We identified the presence of FcγRI, II, IIb and III on RA synovial macrophages and, in addition, expression of FcγRI, FcγRII and FcγRIII revealed by DAB staining was significantly correlated with the expression of the macrophage marker CD163. This indicates that macrophages are likely to be involved in the IC-mediated damage in RA via their FcγR expression and they may also be responsible for antigen presentation to T cells, as macrophages were observed in close proximity to CD3 positive T cells in the RA synovium. The FcγR-positive macrophages were often localised perivasculary together with CD3-positive T cells, most likely as a result of recent extravasation. It is possible that presentation of antigen taken up via FcγRs on the macrophages may activate T cells to secrete cytokines. This could, in turn, lead to further activation of the macrophages and, thus, production of inflammatory cytokines, resulting in a continuous inflammatory state in RA joints.

## Conclusion

Our findings demonstrate that expression of the inhibitory FcγRIIb, as well as of the activating FcγRI, FcγRII and FcγRIII, is increased in RA synovium, regardless of disease duration. The importance of FcγRI and FcγRIIb in the synovial inflammation of RA patients is further highlighted by the fact that healthy synovia lack FcγRI expression and substantially lack FcγRIIb expression. Furthermore, anti-inflammatory drugs, such as glucocorticoids, suppress FcγRI expression after local administration in the joint. These results clearly point towards a central role for the FcγRs in the synovial inflammation of RA.

## Abbreviations

BSA = bovine serum albumin; CCP = citrullinated peptide; CIA = collagen-induced arthritis; DAB = diaminobenzidine; ELISA = enzyme-linked immunosorbent assay; FcγR = Fc gamma receptor; IC = immune complex; ITAM = immunoreceptor tyrosine based activation motif; mAb = monoclonal antibody; PBS = phosphate buffered saline; PE = phycoerythrin; RA = rheumatoid arthritis; RT = room temperature; s = soluble; Sap = saponin.

## Competing interests

Dr Uwe Jacob declares a commercial interest in SuppreMol GmbH. He is one of the cofounders and shareholders of SuppreMol, which develops FcγR agonists and antagonists for clinical use. Dr Uwe Jacob is also listed as inventor on patent applications of SuppreMol regarding the specific FcγRIIb antibodies. The other authors of this paper declare no potential conflicting financial interests.

## Authors' contributions

SK and A-KU were the initiators of the study. SK, SEM, A-KU and ME planned the experiments together. SEM performed all experiments. ME did the tissue sectioning of the biopsies. Immunohistochemistry experiments were performed by SEM with guidance and technical help from ME. SEM and SK did the analysis and evaluation of the immunohistochemistry data. UJ developed the GB3 anti-FcγRIIb specific mAb and contributed with valuable ideas for the study. All authors have read and commented on the text of this paper.
